# Supplementary material for: Targeted Sphingolipid Analysis in Heart, Gizzard, and Breast Muscle in Chickens Reveals Possible New Target Organs of Fumonisins
Source: Toxins (Basel). 2022 Nov 24;14(12):828. doi: 10.3390/toxins14120828 (PMC9783176; doi:10.3390/toxins14120828)
Supplement: Supplementary file 1 [file toxins-14-00828-s001.zip › toxins-2049145 FigureS1-done.pdf]

toxins

MDPI

Article

Targeted Sphingolipid Analysis in Heart, Gizzard, and Breast Muscle in Chickens Reveals Possible New Target Organs of Fumonisins

Philippe Guerre, Caroline Gilleron, Maria Matard-Mann and Pi Nyvall Collén

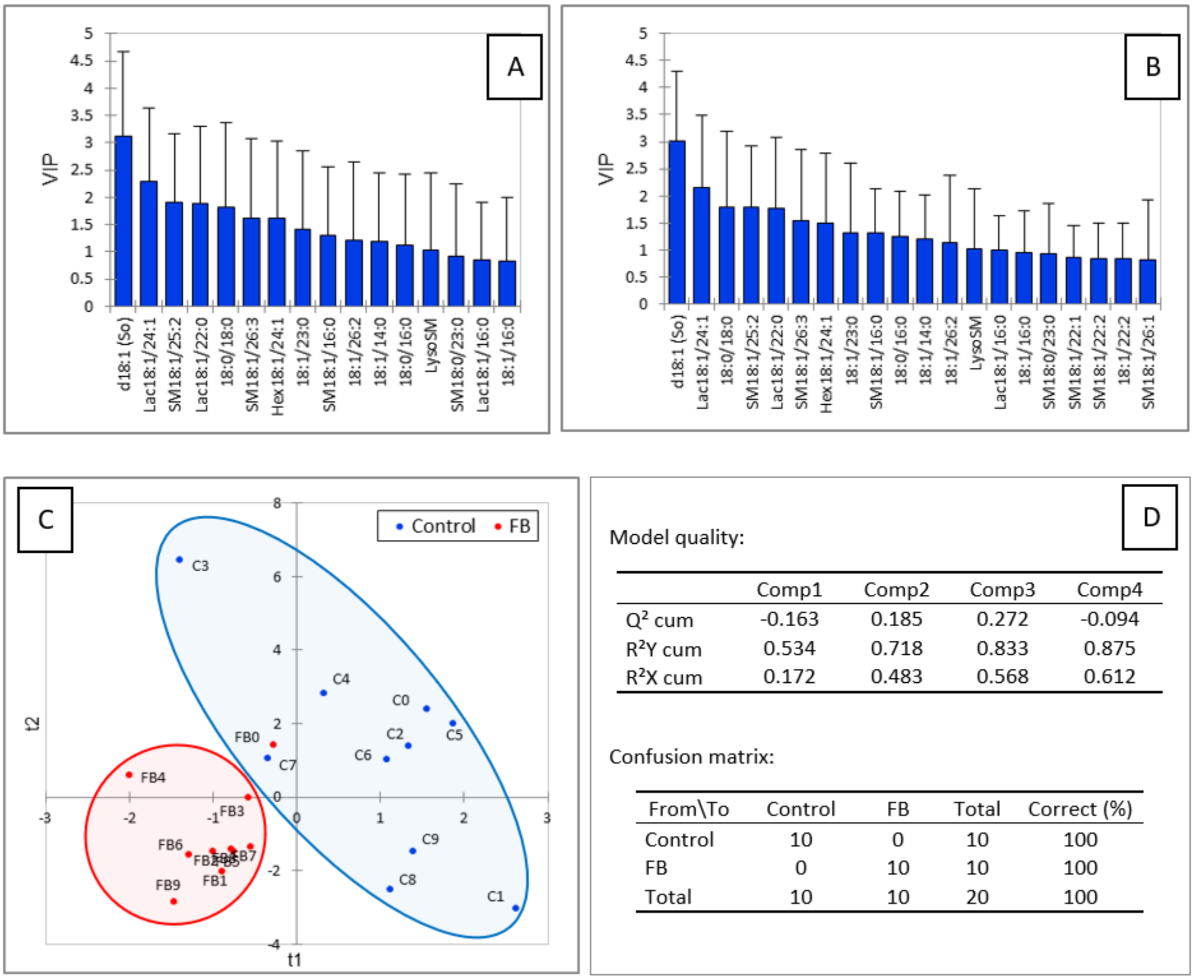

Figure S1. Partial least squares discriminant analysis (PLS-DA) of sphingolipids measured in the breast muscle of chickens fed 9 days with a control diet free of mycotoxins or a diet containing 20.8 mg FB1 + FB2/kg. (A) Scores of variables important in the projection (VIP) for the first component. (B) Scores of variables important in the projection (VIP) for the second component. (C) Discrimination on the factor axes extracted from the original explanatory variables. (D) Quality of the model and confusion matrix for the training sample (variable groups).
